# Stable through the COVID-19 pandemic: Results from a longitudinal telephone interview study in psychiatric outpatients

**DOI:** 10.1371/journal.pone.0276982

**Published:** 2022-11-03

**Authors:** Lisa Kertzscher, Sabrina Baldofski, Elisabeth Kohls, Georg Schomerus, Christine Rummel-Kluge

**Affiliations:** 1 Medical Faculty, Department of Psychiatry and Psychotherapy, Leipzig University, Leipzig, Germany; 2 Department of Psychiatry and Psychotherapy, University Leipzig Medical Center, Leipzig, Germany; The Royal Melbourne Hospital, AUSTRALIA

## Abstract

**Background:**

The COVID-19 pandemic was associated with far-reaching changes all over the world. Health care systems were and are also affected. Little is known about the impact of these changes and the duration of the pandemic on people with mental disorders. The aim of this longitudinal follow-up study was to investigate the mental health status, medical care provision, and attitudes towards the pandemic of these people at the end of the second pandemic lockdown in Germany in 2021, and to compare these findings with the results of 2020.

**Methods:**

People with mental disorders currently receiving treatment in the psychiatric outpatient department of the University Hospital Leipzig, Germany, were asked about depressive symptoms (PHQ-9), self-reported medical care provision, attitudes and social and emotional aspects of the pandemic (social support [ESSI], perceived stress [PSS-4], loneliness [UCLA-3-LS], and resilience [BRS]) using structured telephone interviews.

**Results:**

In total, *N* = 75 participants who had already participated in the first survey in 2020 took part in the follow-up telephone interviews. The most frequent clinician-rated diagnoses were attention deficit disorder/attention deficit hyperactivity disorder (*n* = 21; 28.0%) and obsessive-compulsive disorder (*n* = 16; 21.3%). In comparison to 2020, a significantly higher proportion of participants reported no problems in receiving medical care provision. Compared to the previous year, the resilience of the participants had significantly decreased. Depressive symptoms, social support, perceived stress, and loneliness remained stable. Significantly more participants felt restricted by the pandemic-related government measures in 2021 than in 2020.

**Conclusions:**

This study highlights the importance for continued efforts to maintain stable medical care provision for people with mental disorders during the COVID-19 pandemic, as except for a decrease in resilience, mental health status remained stable. Nonetheless there is still a need for continued treatment to stabilise and improve this status.

## Trial registration

*Trial Registration*: German Clinical Trials Register (DRKS00025112) https://www.google.com/search?client=firefox-b-d&q=DRKS00025112.

## Introduction

The onset of the COVID-19 pandemic in 2020 had an impact on the lives of people around the world. To contain the virus, governmental measures such as lockdown actions and social distancing were taken, which had consequences for public and private life. In addition to the direct and obvious effects of a SARS-CoV-2 infection, there are also important indirect effects of the pandemic on mental health. In comparison to the time before the pandemic, the general population shows lower mental well-being and more symptoms of anxiety and depression [1]. Longitudinal analyses of adults in the UK showed that psychological distress increased one month after the start of the lockdown compared to before the onset of the pandemic [2].

In the face of the COVID-19 pandemic, far-reaching changes took place throughout the health care system and psychiatric care was also subject to pandemic-related changes worldwide (e.g., [3–6]). The importance of giving greater consideration to the possible consequences of contact restrictions on people with mental disorders and facilitating therapeutic services (in accordance with current hygiene regulations) in a timely manner was emphasised (e.g., [7]). In the course of these necessary changes in treatment, remote psychotherapy also became increasingly important. For example, the number of patients receiving psychotherapeutic care in direct, face-to-face contact decreased in Germany, the Czech Republic and Slovakia, and remote psychotherapies increased [8]. In addition, more people received psychotherapeutic treatment online than by telephone [8]. Furthermore, it could be shown that the more people were treated online or via telephone by a therapist, the more positively therapists evaluated their experience with psychotherapy in this form compared to their expectations [9]. Despite all efforts and studies, data is still inconclusive on the impact of the pandemic and related interventions on people with pre-existing mental disorders. In some cross-sectional studies, people with mental disorders show only moderate effects of the pandemic with minor symptom exacerbations (e.g., [10,11]), while other studies found exacerbations of symptoms in more than half of the participants (e.g., [12,13]).

To date, very few longitudinal studies exist on the effects of the pandemic on people with mental disorders. A longitudinal online questionnaire study in the Netherlands involving people with depressive, anxiety, and obsessive-compulsive disorders and without any lifetime mental health disorders concluded that although people with mental disorders had more severe symptoms than people without mental disorders during the COVID-19 pandemic, they did not report a greater increase in symptoms [14]. Rather, people without mental disorders reported a greater increase in symptoms during the pandemic [14]. In contrast, those whose mental health was under the greatest strain even showed a slight decrease in symptoms. A longitudinal study in people with mental disorders in Germany found that most psychiatric symptoms changed only minimally from April/May 2020 to November/December 2020, while resilience decreased [15]. Resilience, as the ability of an individual to withstand stressful life circumstances [16], is of particular interest here. A large body of research has demonstrated the important role that resilience plays in adaptation to adverse circumstances (e.g., [17,18]). Declining resilience, when coping capacities are exhausted, can ultimately lead to a clinical worsening of the symptomatology of people with mental disorders, and this knowledge should also be used in the context of treatment offers [15]. The COVID-19 pandemic represents a stressful event in itself, but also regarding the consequences on everyday lives (e.g., in the areas of employment situation, financial situation, and medical care provision).

Regarding the pandemic situation in Germany, there were two lockdowns. After the first lockdown in March and April 2020, the pandemic situation eased in the summer, however, case numbers rose again from the fall onward. Therefore, a second and longer lockdown lasted from mid-December 2020 until spring 2021 [19]. In May 2021, there were increasing relaxations of restrictions in Germany. During the lockdowns, patient contacts in the psychiatric outpatient department of the University Hospital Leipzig, Germany, were carried out by telephone or online if possible. Group services were adapted to the existing hygiene measures. In addition, new care services were created (e.g., group chats, see [20]).

In 2020, immediately after the first lockdown in Germany, a telephone interview study was carried out in the psychiatric outpatient department of the University Hospital Leipzig [21]. The majority of participating people with mental disorders reported clinically relevant depressive symptoms. It was shown that a low self-rated medical care provision was associated with more pronounced depressive symptoms. Furthermore, higher perceived stress levels were identified as predictors of more depressive symptoms, while social support had no predictive effect [21].

The telephone survey was repeated in the time period beginning 10 weeks during the second lockdown in 2021 and lasting until 2 weeks after the end of the lockdown. The aims of this longitudinal follow-up study were to investigate the mental health status, medical care situation, and attitudes towards the pandemic of people with mental disorders receiving psychiatric outpatient treatment during this time period. Since the same people were interviewed twice, the changes in the abovementioned variables will also be examined.

Based on existing studies, it was hypothesized that due to the duration of the pandemic and the restrictions in place, and despite efforts to maintain continued medical care provision, the mental health status of the interviewed people would have decreased and their perceived stress increased. In addition, it was assumed that a certain "pandemic fatigue", a general state of mental exhaustion, which is also associated with a decrease in motivation to comply with recommended measures [22], would be visible in a decreased resilience.

## Materials and methods

### Participants and procedure

Structured telephone interviews with psychiatric outpatients currently receiving treatment in the psychiatric outpatient department of the University Hospital Leipzig, Germany were conducted between April 26 and July 15, 2021. The same inclusion and exclusion criteria and procedures applied as in the first survey in 2020 (for details see [21]). Before conducting the interviews, each participant’s verbal informed consent was audio recorded by the interviewer via telephone and also separately documented in digital form. Two independent and trained psychologists conducted the structured interviews and entered the data at the same time (survey tool Questback). This survey method was chosen due to applicable pandemic measures, because no face-to-face interviews were possible due to the contact restrictions. Nevertheless, personal contacts via telephone were chosen in order to obtain as many participants as possible, as it is known that response rates in online surveys are generally lower than in telephone interviews [23]. In addition, informed consent was collected and recorded verbally to make the process simple for participants and to obtain the informed consent, as face-to-face contacts were not possible due to pandemic measures. As people with mental disorders are a highly loaded target group and in order to have as few missing values in the responses as possible, it was decided to conduct telephone interviews with validated questionnaires. The measurement instruments used are validated and were read out with the given answer format, therefore rater differences could be excluded. The mean duration of the telephone interviews was 48.85 (*SD* = 24.36) minutes.

The first telephone interview survey in 2020 was completed by *n* = 106 people. For this longitudinal follow-up study, *n* = 4 participants were not contacted again for the second interview in 2021 due to difficulties during the first interview (e.g., language barrier, lack of compliance to answer questions). Thus, a total of *n* = 102 people were contacted by telephone. Of these, *n* = 75 (73.5%) participated in the second telephone interview, while *n* = 11 (10.8%) declined to participate, *n* = 11 (10.8%) could not be reached, and *n* = 5 (4.9%) were interested in participating, but ultimately did not take part.

The research project was approved by the Ethical Committee at the Medical Faculty, Leipzig University (April 13, 2021, 174/21-ek). The study was registered at the German Clinical Trials Register (DRKS00025112).

### Measures

For the telephone interviews, a slightly modified version of the first questionnaire was used [21]. As in the first survey, the participants were asked for socio-demographic information (age, gender, marital status, being parent, residential status, migration status). The main clinician-rated diagnosis for which a patient was currently receiving treatment was assessed.

In addition to the first survey, the participants were asked about their evaluation of the second lockdown compared to the first lockdown, as well as their COVID-19 vaccination status and their attitude towards vaccination, and changes in employment and income situation since the beginning of March 2020.

Further, the following assessments were conducted (for details please see [21]):

#### COVID-19 pandemic and lockdown: Attitudes, restrictions, and self-rated medical care provision

Identical with the first telephone interview, items adapted to the pandemic situation from an existing cohort study questionnaire were used [24] and comprised the following aspects (for details please see [21]):

Participants’ attitudes towards the COVID-19 pandemic, the lockdown and the governmental measures and restrictions related to the second lockdown as well as the individual impact of the measures on different areas of life, self-reported medical care provision (overall, general practitioner, psychiatrist, psychotherapist, psychiatric outpatient department) and adherence to and mode of medical appointments during the second lockdown (multiple choice), as well as positive and negative aspects of the pandemic (open-ended question) were explored. Additionally, it was assessed whether the participant or someone in their family or acquaintances had been infected with COVID-19 at the time of the telephone interviews or before, or had been in quarantine at the time of the interviews or in the three months before.

#### Depressive symptoms, social and emotional aspects of the COVID-19 pandemic and lockdown

To measure depressive symptoms over the last 14 days the **Patient Health Questionnaire-9** (**PHQ-9**; [25]) was administered. A sum score of 5 is the lower limit of mild depression, a score of 10 of moderate, a sum score of 15 of moderately severe depression, and a score of 20 of severe depression.

The perceived social support was assessed using the German adaptation of the **Enriched Social Support Inventory** (**ESSI**; [26]).

To measure subjectively perceived loneliness and social isolation, a German adaptation of the **UCLA 3-Item loneliness scale** (**UCLA-3-LS**; [27]) was used.

The **Brief Resilience Scale** (**BRS**; [28]) was administered to measure resilience.

The perceived stress level over the last month was assessed using the **Perceived Stress Scale 4** (**PSS-4**; [29]).

### Statistical analysis

All analyses were conducted using IBM SPSS statistics version 27.0. A two-tailed α = 0.05 was applied to statistical testing. In order to rule out a potential attrition bias, participants who had only taken part in the 2020 survey and participants who completed both measurement points were examined with regard to differences in baseline sociodemographic variables. A *t*-test for continuous variables (age) and chi-square tests for categorical variables (gender, marital status, psychiatric diagnosis) were used.

First, a descriptive analysis for the total sample was performed, including the following variables: sociodemographic information; medical care provision; PHQ-9, PSS-4, ESSI, UCLA-3-LS, and BRS scores; adherence to and mode of medical appointments; attitudes towards the pandemic; positive and negative aspects of the pandemic; information on corona infections and vaccinations; and lifestyle (healthy diet, physical activities, social activities) and related restrictions. The answers to the open-ended questions were divided into categories using a content analysis approach, i.e., answers with similar content were assigned to one category, and then analyzed descriptively. All participants were included in this analysis. Due to missing values, analyses on appointments with a psychiatrist, healthy diet and physical activities included reduced sample sizes of *n* = 74, *n* = 71 and *n* = 72, respectively.

Second, differences between both surveys from 2020 and 2021, respectively, were investigated regarding the following variables: BRS, medical care provision, PHQ-9, PSS-4, ESSI, UCLA-3-LS, restrictions due to the pandemic, and attitudes towards the pandemic. Regarding the analyses on continuous variables, only the BRS scores were normally distributed, as assessed by the Shapiro-Wilk test (*p* > .05). Therefore, a paired *t*-test was calculated to assess differences in BRS scores. Effect size was interpreted as suggested by Cohen [30].

Due to the non-normal distribution of all other continuous variables (as indicated by Shapiro-Wilk tests, all *p* < .05), Wilcoxon signed-rank tests were calculated to compare the self-rated medical care provision, the scores of PHQ-9, PSS-4, ESSI, and UCLA-3-LS, and the changes in self-reported restrictions due to the COVID-19 pandemic between 2020 and 2021. A symmetrical distribution of values was given by visual inspection of the histogram of difference scores. For the self-rated medical care provision and the PHQ-9, the number of bars was calculated according to Freedman and Diaconis [31] and the symmetrical distribution of the differences was additionally checked manually and could be confirmed.

For categorical variables, McNemar tests were calculated to determine differences in attitudes towards the COVID-19 pandemic. For this analysis, all items on attitudes towards the COVID-19 pandemic were first dichotomised (0 = disapproval, 1 = agreement).

For Wilcoxon signed-rank tests and McNemar tests, Bonferroni corrections were applied to counteract the alpha error cumulation.

To examine longitudinal effects in the change of depressive symptoms, a linear regression analysis was performed. In addition to comparing means, this analysis allows to consider the magnitude of dispersion in the change in sum scores of depressive symptoms. For this analysis, the following variables were used: gender, age, educational level, marital status, residential status, being parent, self-rated medical care provision, depressive symptoms (PHQ-9 sum score), perceived stress (PSS-4 sum score), social support (ESSI sum score), loneliness (UCLA-3-LS sum score), and resilience (BRS sum score). Sociodemographic data were used from the 2021 dataset. Categorical variables were dichotomized (educational level, marital status, residential status). For the variables self-rated medical care provision, depressive symptoms, perceived stress, social support, loneliness, and resilience differences between the two measurement timepoints were calculated. The difference in depressive symptoms was used as dependent variable. As predictor variables the sociodemographic variables, the sum scores and self-rated medical care provision of the baseline measurement and the calculated differences of these variables were used. All predictor variables were inserted into the model simultaneously. Data were checked for outliers, normal distribution of variables, and residuals. Homoscedasticity could be expected and there was no multicollinearity (Variation Inflation Factor [VIF] ≤ 10; correlation matrix check, *r* ≤ .85).

## Results

### Sample characteristics

Overall, *N* = 75 participants (female: *n* = 36 [48.0%]; male: *n* = 39 [52.0%]) took part in the telephone interviews, with an age ranging between 20 and 79 years (*M* = 39.11, *SD* = 13.1; see Table 1). The most frequent clinician-rated diagnoses (main diagnosis) were attention deficit disorder/attention deficit hyperactivity disorder (ADD/ADHD; *n* = 21, 28.0%), obsessive-compulsive disorder (OCD; *n* = 16, 21.3%), and anxiety disorder (*n* = 10, 13.3%).

**Table 1:** Sample characteristics.

	Second interview 2021 (*N* = 75)
**Gender**, *n* (%)	
Female	36 (48.0)
Male	39 (52.0)
**Age**, *M (SD*); years	39.11 (13.1)
**Marital status**, *n* (%)	
Married	22 (29.3)
**Residential status**, *n* (%)	
Alone^a^	30 (41.1)
With partner/spouse^b^	31 (41.9)
With children^b^	14 (18.9)
**Migration**, *n* (%)	
Self	4 (5.3)
Parents	4 (5.3)
**Being Parent**, *n* (%)	
Yes	29 (38.7)
**Psychiatric comorbidity status**, *n* (%)	
Yes	36 (48.0)
**Mental disorder, clinician-rated diagnosis** [*n* (%); main diagnosis]	
ADD/ADHD	21 (28.0)
OCD	16 (21.3)
Anxiety disorder	10 (13.3)
Unipolar depression	7 (9.3)
Bipolar depression	6 (8.0)
Schizophrenia and delusional disorder	6 (8.0)
Personality disorder	2 (2.7)
Eating disorder	2 (2.7)
Schizoaffective disorder	2 (2.7)
PTSD	1 (1.3)
Other	2 (2.7)

^a^ sample size *n* = 73 due to missing data.

^b^ sample size *n* = 74 due to missing data.

ADD/ADHD = attention deficit disorder/attention deficit hyperactivity disorder.

OCD = obsessive-compulsive disorder.

PTSD = post-traumatic stress disorder.

*N* = total population.

*n* = sample size.

*M* = mean value.

*SD* = standard deviation.

There were no significant differences in age, *t*(104) = 1.334, *p* = .185, gender, **χ²(1) = 3.000,** *p* **= .083**, marital status, **χ²(1) = 0.134,** *p* **= .714**, and psychiatric diagnosis**,** χ²(10) = 5.362, *p* = .866, between participants who only took part in the 2020 survey or both the 2020 and 2021 surveys.

### Medical care provision, depressive symptoms, and social and emotional aspects of the COVID-19 pandemic and lockdown

Almost three quarters of the interviewed participants (*n* = 53, 70.7%) reported that they had no difficulties receiving medical care provision during the second lockdown and that all scheduled treatments had taken place, compared to less than half a year earlier (*p* = .005, see Table 2). Interviewed people with mental disorders reported to have had statistically significant lower difficulties in receiving medical care provision in 2021 than in 2020. Specifically, more than half of the interviewed people (*n* = 45, 60.0%) were able to keep their appointments with their general practitioner. The same held true for appointments with a psychiatrist (*n* = 28, 37.8%), in the psychiatric outpatient department (*n* = 27, 36.0%), and with a psychotherapist (*n* = 15, 20.0%).

**Table 2:** Medical care provision, depressive symptoms, and social and emotional aspects (2020 and 2021).

	First interview 2020 (*N* = 75)	Second interview 2021 (*N* = 75)	Mdn 2020	Mdn 2021	*T*	Standardised *z*	*p*	Effect size
**Self-rated medical care provision**: *M (SD)*	1.87 (1.08)	1.45 (0.89)	2.00	1.00	252.000	-2.800	**.005**	*r* = .32
**Depressive Symptoms**, PHQ-9; *M (SD)*	10.68 (5.71)	11.16 (5.45)	9.00	10.00	1384.500	1.061	.289	*r* = .12
**Perceived Stress**, PSS-4; *M (SD)*	8.15 (3.65)	7.83 (3.40)	8.00	8.00	1015.000	-.777	.437	*r* = .09
**Social Support**^**a**^, ESSI; *M (SD)*	21.03 (3.63)	20.36 (3.79)	22.00	21.00	660.000	-1.523	.128	*r* = .18
**Loneliness**^**a**^, UCLA-3-LS; *M (SD)*	5.22 (1.99)	5.52 (1.69)	5.00	5.00	650.000	-1.828	.068	*r* = .18
**Resilience**^**a**^, BRS; *M (SD)*	2.84 (.96)	2.63 (.97)	-	-	2.058	-	**.043**	*d* = .24

^a^ First interview 2020: Sample size *n* = 74 due to missing data.

PHQ = Patient Health Questionnaire-9 sum score.

Perceived Stress = Perceived Stress Scale-4 sum score.

Social Support = ENRICHED Social Support Inventory sum score.

Loneliness = University of California Los Angeles Loneliness Scale sum score.

Resilience = Brief Resilience Scale sum score.

*N* = total population.

*M* = mean value.

*SD* = standard deviation.

Mdn = Median.

*T* = test statistics.

*z* = standardised value.

*r* = correlation coefficient, effect size for Wilcoxon signed-rank test.

*d* = effect size for *t*-test.

Almost all participants (*n* = 50, 98.0%) stated that they had attended their appointments with their general practitioner in person. Likewise, about three quarters of the interviewed people attended their appointments with the psychiatrist in person (*n* = 51, 73.9%), the psychiatric outpatient department (*n* = 33, 78.6%) and with the psychotherapist (*n* = 22, 78.6%). Appointments by telephone were most common with the psychiatrist (*n* = 38, 55.1%), followed by appointments with the psychotherapist (*n* = 9, 32.1%), the psychiatric outpatient department (*n* = 3, 7.1%) and the general practitioner (*n* = 1, 2.0%). Video consultations were used only sporadically (psychotherapist: *n* = 4, 14.3%; psychiatrist: *n* = 3, 4.3%, psychiatric outpatient department: *n* = 1, 2.4%). Some participants (*n* = 3, 7.1%) reported that they participated in new treatment offers (e.g., group or chat offers) created in the psychiatric outpatient department during the pandemic.

In the current survey, the interviewed people also had clinically relevant moderate depressive symptoms, which were slightly more pronounced than in 2020. This change was not statistically significant (*p* = .289, see Table 2). There were also minimal changes in the other scales. However, these differences were not statistically significant. Perceived stress had decreased slightly in 2021 compared to 2020 (*p* = .437), as had perceived social support (*p* = .128). Perceived loneliness had increased slightly in 2021 compared to 2020 (*p* = .068). A paired t-test revealed a significantly lower BRS score in 2021 than in 2020, *t*(73) = 2.058, *p* = .043.

### Attitudes towards the COVID-19 pandemic

Almost all participants (*n* = 71, 94.7%) denied having been infected with the coronavirus at some point or at the time of the telephone interviews. More than half of the interviewed participants knew people from their family or acquaintances who had been infected with the virus at the time of the interviews or before (*n* = 42, 56.0%). Less than one-fifth (*n* = 11, 14.7%) reported knowing one or more persons who had died from COVID-19.

Almost all participants (*n* = 71, 94.7%) were not in quarantine at the time of the telephone interviews or in the three months before. A small proportion (*n* = 11, 14.7%) reported that someone in their household or own family had been in quarantine during this period.

A minority (*n* = 2, 2.7%) considered their own risk of becoming infected with COVID-19 to be extremely likely at the time of the telephone interviews. ‘Extremely unlikely’ or ‘unlikely’ was stated by 64.0% (*n* = 48). Of the interviewed people, *n* = 68 (90.7%) reported to be somewhat or completely in favour of vaccination in general. At the time of the telephone interviews, just under two-thirds of participants (*n* = 49, 65.3%) had received medical advice on COVID-19 vaccinations and about half of the participants (*n* = 40, 53.3%) indicated that they had already been vaccinated against COVID-19.

More than half of the participants (*n* = 49, 65.3%) stated that there had been no major changes in their employment situation since the start of the first lockdown beginning in March 2020 in Germany, and more than half of the participants (*n* = 48, 64.0%) reported that there had been no major changes in their financial situation, too. About half of the interviewed people (*n* = 36, 48.0%) indicated that the second lockdown was more difficult and worse compared to the first lockdown. In contrast, about one fifth of the participants (*n* = 14, 18.7%) reported that the second lockdown was easier and better for them than the first lockdown.

Almost all participants (*n* = 72, 96.0%) named negative aspects of the corona pandemic and the related measures. The most frequently mentioned negative aspects were the restrictions in the social, cultural, and sporting areas (*n* = 46, 61.3%), followed by the negative impact on jobs, studies, and school (*n* = 19, 25.3%), and on medical care and mental health (*n* = 15, 20.0%). Positive aspects were mentioned by *n* = 47 (62.7%). The most frequently mentioned positive impact was social, such as less social pressure, more solidarity and more time for family and friends (*n* = 13, 17.3%), followed by the possibility to work from home and a better work/study/life balance (*n* = 12, 16.0%), and deceleration of the everyday life as a result of the pandemic and restrictions (*n* = 10, 13.3%).

Regarding the attitudes, the vast majority of participants agreed to be optimistic about coming through the corona crisis unscathed (*n* = 69, 92.0%, see S1 Table), that people with mental disorders would suffer particularly badly from the corona crisis (*n* = 69, 92.0%), and that they would fully support the government’s measures to contain the virus (*n* = 68, 90.7%). Compared to 2020, the proportion of those who were optimistic about coming through the corona crisis unscathed had increased (*p* = 0.41, not significant after Bonferroni correction). As in 2020, more than half of the participants (*n* = 44, 58.7%) responded in 2021 that they were experienced in crisis management due to the mental disorder and therefore could cope better with the situation than many other people. There was a statistically significant change in one attitude towards the COVID-19 pandemic between 2020 and 2021: In 2021 more participants felt severely restricted by the government measures to slow down the coronavirus than in 2020 (*p* = .003, Bonferroni-corrected).

Regarding the lifestyle and self-reported pandemic related restrictions, three quarters of the participants (*n* = 53, 74.6%) did not feel restricted in their healthy diet due to the pandemic and related measures. More than three quarters of the interviewed people (*n* = 56, 77.8%) felt a little or strongly restricted in their physical activities and the majority of the participants (*n* = 69, 92.0%) felt restricted in their social activities. In comparison with the results from 2020, significant differences were only found regarding restrictions on physical activities. Interviewed people reported significantly higher perceived restrictions in their physical activities in 2021 (*Mdn* = 1.0) than in 2020 (*Mdn* = 1.0), *T* = 530.0, *z* = 2.427, *p* = .015 (Bonferroni-corrected).

### Longitudinal changes in depressive symptoms

To examine longitudinal effects in the change of depressive symptoms between 2020 and 2021, a linear regression analysis was performed (see Table 3). The *R*² for the overall model was 0.61, *F*(17, 69) = 4.81, *p* < .001. Significant effects were shown for the following variables: PHQ-9 sum score of 2020 (baseline), PSS-4 sum score of 2020 (baseline), the change in PSS-4 sum scores between the two measurement time points (perceived stress difference), and the change in BRS sum score between the two measurement time points (resilience difference). Specifically, these significant predictive effects indicate the following: The higher the depressive symptom scores in 2020, the lower the increase in depressive symptoms from 2020 to 2021 (*p* < .001). Further, a higher perceived stress level at baseline was associated with a greater increase in depressive symptoms (*p* = .017). In addition, it was found that the greater the increase in perceived stress, the greater the increase in depressive symptoms (*p* < .001). Furthermore it was shown that the greater the decrease in resilience, the greater the increase in depressive symptoms (*p* = .045). All other predictor variables did not show significant effects (all *p* > .05).

**Table 3:** Regression analysis on longitudinal changes in depressive symptoms (*n* = 69).

Variable	Unstan-dardized B	SE B	Standar-dized β	95% Confidence Interval (CI)		*t*	*p*
Gender	0.15	1.14	0.02	-2.13	2.43	0.13	.896
Age	-0.01	0.05	-0.03	-0.11	0.09	-0.22	.827
Educational level	-0.89	1.38	-0.08	-3.65	1.88	-0.64	.522
Marital status	0.83	1.47	0.08	-2.12	3.79	0.57	.574
Residential status	-0.53	1.18	-0.05	-2.90	1.84	-0.45	.655
Being parent	-2.01	1.37	-0.19	-4.76	0.73	-1.47	.147
Self-rated medical care provision 2020	1.56	1.10	0.24	-0.65	3.77	1.42	.161
Self-rated medical care provision difference	0.52	0.85	0.10	-1.18	2.22	0.61	.542
Depressive symptoms (PHQ-9) 2020	-0.66	0.12	-0.74	-0.91	-0.42	-5.52	**< .001**
Perceived stress (PSS-4) 2020	0.61	0.25	0.43	0.12	1.11	2.47	**.017**
Perceived stress (PSS-4) difference	0.64	0.17	0.45	0.29	0.99	3.69	**< .001**
Social support (ESSI) 2020	-0.28	0.16	-0.19	-0.60	0.04	-1.76	.085
Social support (ESSI) difference	-0.20	0.18	-0.13	-0.56	0.16	-1.10	.276
Loneliness (UCLA-3-LS) 2020	0.16	0.34	0.06	-0.53	0.85	0.46	.648
Loneliness (UCLA-3-LS) difference	0.20	0.37	0.06	-0.55	0.95	0.54	.590
Resilience (BRS) 2020	0.18	0.83	0.03	-1.48	1.84	0.22	.828
Resilience (BRS) difference	-1.45	0.71	-0.26	-2.87	-0.03	-2.05	**.045**
*F*	4.81						
*R² (R² adjusted)*	0.61 (0.48)						
*p*	< .001						

Perceived Stress = Perceived Stress Scale-4 sum score.

Social Support = ENRICHED Social Support Inventory sum score.

Loneliness = University of California Los Angeles Loneliness Scale sum score.

Resilience = Brief Resilience Scale sum score.

*n* = sample size.

B = regression coefficient.

SE B = standard error regression coefficient.

standardized β = standardized regression coefficient.

CI = Confidence Interval.

*t* = test statistics.

*F* = measure of variance ratio.

*R² =* multiple determination coefficient.

## Discussion

The aim of this telephone survey was to investigate longitudinal changes in the attitudes, depressive symptoms, and social and emotional aspects of people with mental disorders in a psychiatric outpatient department of a German university hospital after one year of the COVID-19 pandemic.

The results of the two surveys were comparable: Depressive symptoms, perceived stress, social support, and loneliness had remained stable. The severity of the depressive symptoms was in the medium clinically relevant range. Thus, the initial hypothesis of a general deterioration of the mental health status and an increased stress experience by the respondents cannot be confirmed. The participants’ resilience had significantly decreased after one year, whereas the perceived medical care provision had significantly improved compared to 2020. Further, significantly more participants reported feeling constrained by pandemic-related government measures in 2021 than in 2020. These results, the decrease in resilience and the increased experience of limitations due to the pandemic measures, correspond with the hypothesis that after more than one year of the COVID-19 pandemic, a kind of "pandemic fatigue", a general state of mental exhaustion, set in among the respondents. As a further result, perceived restrictions on physical activities were also significantly greater in 2021 than in 2020. In line with the results, it could be shown in the longitudinal course that the initial levels of depressive symptoms and perceived stress levels are associated with the increase in depressive symptoms. In addition, significant influences on the increase in depressive symptoms were shown in regards to the changes that took place between the two measurement time points. Specifically, the greater the increase in perceived stress between the two years, the greater the increase in depressive symptoms, and the greater the decrease in resilience, the greater the increase in depressive symptoms.

It is known that in the context of the COVID-19 pandemic, it is not only people with mental disorders who have reported a worsening of their mental health status [1]. The general population also shows lower mental well-being and more severe symptoms of anxiety and depression than before the pandemic [1]. It was shown that the second wave of the COVID-19 pandemic is having a negative impact on mental health [32], and COVID-19-related mental health problems can pose a major threat to the general population [33]. In addition, after more than one year of the pandemic and two lockdowns, mental distress in Germany remains high, depression symptoms increased, while pandemic fatigue set in and safety behaviour decreased during the second lockdown [22]. Early on in the pandemic, the importance of giving greater consideration to people with mental disorders in the context of contact restrictions and also enabling therapeutic services in a timely manner in accordance with applicable regulations was emphasised (e.g., [7,20]). In this context, the potential of e-mental health services was also repeatedly highlighted (e.g., [34]).

But could these recommendations also be transferred to everyday clinical practice? At the end of the second lockdown in 2021 in Germany, significantly more people with mental disorders indicated in the telephone interviews that they had fewer or no problems receiving medical care provision than in 2020. In compliance with applicable hygiene measures, the majority of appointments in the psychiatric outpatient department could be attended in person. Through new care offers, such as group chats, an adaptation of existing offers (e.g., smaller groups, outdoor walk-and-talk groups), and an improvement of the technical equipment (e.g., online individual appointments), it was possible to maintain more treatment offers than during the first lockdown, despite existing restrictions and to complement existing offers. While the first lockdown was mainly about adapting to the new and dynamic situation, it was possible to draw on initial experience during the second lockdown.

Already during the first survey in 2020, half of the participants had stated that they were better able to deal with the situation than many other people due to their mental disorder and the associated experience in crisis management [21]. The proportion of those who agreed to this statement had even slightly increased in 2021. In addition, the psychometric measures on depressive symptoms, perceived stress, social support, and loneliness showed that the respondents had mastered the time period between the first and second survey in a stable manner. Only resilience had significantly deteriorated after one year. In the course of the pandemic a decrease in resilience has already been described in another study (see [15]). Resilience describes a person’s ability to withstand stressful life circumstances [16]. The results of the interviews indicate that the participants were able to buffer the potential negative consequences of the lockdown on their mental health relatively well, despite their reduced resilience. However, all the changes undertaken in the treatment offers cannot completely compensate for the duration of the pandemic and the lack of perspective. Thus, the decreased resilience can also be interpreted as a kind of pandemic fatigue, which is also shown in the finding that, compared to 2020, significantly more participants agreed that they feel severely restricted by the government measures, and these restrictions are also evident in everyday life, in the area of physical activities. The forces required to adapt to ever-changing circumstances are dwindling. The outcome of reduced resilience is also important because in this study the greater the decrease in resilience, the greater the increase in depressive symptoms. In line with this it is known from the literature that a decrease in coping strategies can ultimately result in clinical worsening of symptoms in people with mental disorders [15]. Thus, the decreased resilience can also be considered as a warning signal to prevent symptom deterioration to the point of inpatient treatment.

A possible reason for the stable mental health symptoms might be the quality of social contacts, despite the pandemic-related reduction in the quantity of social contacts. It has been shown that the quality and quantity of social relationships, among other aspects, influence mental health [35]. An Austrian survey at the end of the first lockdown emphasised the importance of social relationships in promoting resilience by mitigating negative physical and psychological health consequences [36]. Because participants’ social support had not changed significantly, social contacts may have acted as a buffer to counteract further deterioration in resilience and overall mental health status. In our study, more than half of the participants have stated that they do not live alone, which ensures social contacts even in times of contact restrictions. In addition, the treatment offers in the psychiatric outpatient clinic were constantly adapted to existing arrangements, thus ensuring ongoing contact with treatment providers, which is also important for the day-structuring function, as routines are crucial for the stability and level of functioning of people with mental disorders [37].

Since it is unclear how long the COVID-19 pandemic will continue to occupy the people to the present extent and with these far-reaching effects on their lives, the telephone interviews also provide important starting points for further adjustments in treatment services. Changes already undertaken seem to have been well accepted by the people with mental disorders and should be further established and continued. In addition, it would be a possibility to integrate psychoeducational groups on resilience and self-care into the treatment, as a decreased resilience between 2020 and 2021 could be found in the present study. These could also be carried out online, and already existing e-mental health programmes offer many possibilities in this regard. Even before the pandemic, there were apps and programmes aimed at mental health and resilience (e.g., Woebot Health [38]; Resilience App [39]). In the wake of the pandemic, there have been new developments especially aiming at building and improving psychological resilience during the pandemic within the general population (e.g., BOUNCE–Building Up Your Resilience; [40]). There are many more opportunities here to adapt these offers to specific target groups (e.g., for people with mental disorders or young people). E-mental health services should play a key role in this, and the COVID-19 pandemic could thus represent a turning point for e-mental health (e.g., [34]). As another result of the study, physical activities can also be more focused on, according to applicable government pandemic measures. In the context of psychiatric treatment, smaller groups and activities that take place outdoors could complement the possibilities of e-mental health. In addition, wearable technology could play an important role in increasing physical activity, especially in everyday life, as studies with various samples are yielding promising initial results (e.g., [41–43]).

In summary, many efforts have already been made to ensure treatment for people with mental disorders during the COVID-19 pandemic and those affected seem to benefit from these efforts and services. However, the interviewed people still report clinically relevant depressive symptoms and need ongoing medical care provision. Therefore, further adaptations to the changing needs of the target group are necessary and worthwhile, and the interviews conducted in this study provided some starting points.

### Strengths and limitations

One of the strengths of the telephone interview study is the sample size and the longitudinal design. Of the *n* = 106 participants from the first telephone study in 2020, *n* = 75 participants could be won over for repeated participation in 2021. This is particularly important due to the length of the telephone interviews. Thus, a unique insight into the state of mind of people with mental disorders in the time period beginning at the end of the second lockdown was possible. But a comparison can also be made with the situation immediately after the first lockdown in 2020. In addition, the interviews were again conducted by trained psychologists, which can be considered more elaborate than using questionnaires.

In addition to the strengths mentioned above, there are a number of limitations that should be taken into account when interpreting the results. A limitation of the study is that the survey took place in the time period beginning at the end of the second lockdown. As regulations has changed during conducting the interviews there is a variance in the environmental conditions of the participants at the beginning and end of the interview period. In addition, interview participants in telephone interviews tend to be more socially desirable in their responses and suspicious of the interview process compared to face-to-face interviews [44]. This should be taken into account when interpreting the results. Moreover, as the recruitment took place in only one psychiatric outpatient department, it was not possible to cover all social minorities within the study sample. To increase generalizability of the findings, future studies could examine the assessed variables in samples including social minorities, e.g., in homeless people or people with minoritized sexual and gender identities. Another limitation is the small sample size of the linear regression analysis. The analysis was performed to detect longitudinal effects of depressive symptoms. The limited power of the analysis due to the small sample size and the number of predictor variables must be kept in mind when interpreting the results. Furthermore, in the present study, the most common disorders are ADD/ADHD and OCD. This is a special characteristic of the sample studied and should be taken into account when generalizing the results. In particular, regarding the diagnosis of ADHD and OCD in adults, there is a need for awareness on the part of treatment providers. There is still a discrepancy between the prevalences resulting from epidemiological studies and the prevalence of actual ADHD and OCD diagnoses (e.g., [45,46]). Since the clinic at which the survey was conducted has specializations in this area, these diagnoses occur more frequently in the sample studied than in other samples of psychiatric outpatients.

## Conclusion

In summary, people with mental disorders receiving outpatient treatment have been affected by the COVID-19 pandemic and the resulting government policies and changes in Germany. During the pandemic and the first and second lockdown, a number of measures have been taken to maintain treatment in spite of restrictions in place.

Despite increased and noticeable restrictions due to the measures and a decreased resilience, participants’ mental health symptoms remained stable, but they still reported clinically relevant depressive symptoms. Adjustments made in the treatments seem to be effective and largely counteract an acute worsening of symptoms. The participants’ own experiences in crisis management also come into play.

However, constant changes and adaptations in the care system are necessary to keep the condition of those affected stable and to improve it. People with mental disorders and their ongoing treatment must continue to be considered in the pandemic measures. It is thanks to many dedicated treatment providers from different professional groups that such an outcome could be achieved. This study provides a snapshot. Further research is needed to continue to ensure, optimise, and adapt medical care for people with mental disorders to changing conditions.

## Supporting information

S1 Table Agreement to statements regarding the pandemic (McNemar tests; *N* = 75). (DOCX)
