# Supplementary material for: Stable through the COVID-19 pandemic: Results from a longitudinal telephone interview study in psychiatric outpatients
Source: PLoS One. 2022 Nov 3;17(11):e0276982. doi: 10.1371/journal.pone.0276982 (PMC9632763; doi:10.1371/journal.pone.0276982)
Supplement: S1 Table — (DOCX) [file pone.0276982.s001.docx]

**S1 Table. Agreement to statements regarding the pandemic (McNemar tests; *N* = 75).**

| **Statement (Attitude)** | **2020**  ***n* (%)** | **2021**  ***n* (%)** | ***p*** |
| --- | --- | --- | --- |
| I am worried about the coronavirus. | 46 (61.3) | 48 (64.0) | .839 |
| I feel personally endangered by the coronavirus. | 25 (33.3) | 33 (44.0) | .115 |
| I fully support the government measures to slow down the spread of the coronavirus. | 64 (85.3) | 68 (90.7) | .219 |
| I feel severely restricted by the government measures to slow down the coronavirus. | 18 (24.0) | 33 (44.0) | **.003** |
| I am optimistic that I will come through the corona crisis unscathed. | 59 (78.7) | 69 (92.0) | .041 |
| People with mental disorders suffer particularly badly from the corona crisis. | 62 (82.7) | 69 (92.0) | .167 |
| The measures to contain the crisis hit people with mental disorders particularly hard. | 59 (78.7) | 64 (85.3) | .332 |
| Because of my mental disorder, I feel more excluded than other people in the corona crisis. | 19 (25.3) | 24 (32.0) | .359 |
| Overall, it is good for me that I do not have to go outside so much and have less contact with other people. | 26 (34.7) | 25 (33.3) | 1.000 |
| Because the crisis affects everyone equally, I feel less excluded than usual. I can bear my disorder better at the moment because I know that all the other people have to live with restrictions too. | 33 (44.0) | 27 (36.0) | .345 |
| The corona crisis and the resulting restrictions have exacerbated my problems. | 35 (46.7) | 40 (53.3) | .332 |
| Because I have learned to deal with crises through my disorder, I can cope with the current situation better than many other people. | 38 (50.7) | 44 (58.7) | .286 |
